# The Landscape of Participatory Surveillance Systems Across the One Health Spectrum: Systematic Review

**DOI:** 10.2196/38551

**Published:** 2022-08-05

**Authors:** Carrie McNeil, Sarah Verlander, Nomita Divi, Mark Smolinski

**Affiliations:** 1 Ending Pandemics San Francisco, CA United States

**Keywords:** participatory surveillance, One Health, citizen science, community-based surveillance, infectious disease, digital disease detection, community participation, mobile phone

## Abstract

**Background:**

Participatory surveillance systems augment traditional surveillance systems through bidirectional community engagement. The digital platform evolution has enabled the expansion of participatory surveillance systems, globally, for the detection of health events impacting people, animals, plants, and the environment, in other words, across the entire One Health spectrum.

**Objective:**

The aim of this landscape was to identify and provide descriptive information regarding system focus, geography, users, technology, information shared, and perceived impact of ongoing participatory surveillance systems across the One Health spectrum.

**Methods:**

This landscape began with a systematic literature review to identify potential ongoing participatory surveillance systems. A survey was sent to collect standardized data from the contacts of systems identified in the literature review and through direct outreach to stakeholders, experts, and professional organizations. Descriptive analyses of survey and literature review results were conducted across the programs.

**Results:**

The landscape identified 60 ongoing single-sector and multisector participatory surveillance systems spanning five continents. Of these, 29 (48%) include data on human health, 26 (43%) include data on environmental health, and 24 (40%) include data on animal health. In total, 16 (27%) systems are multisectoral; of these, 9 (56%) collect animal and environmental health data; 3 (19%) collect human, animal, and environmental health data; 2 (13%) collect human and environmental health data; and 2 (13%) collect human and animal health data. Out of 60 systems, 31 (52%) are designed to cover a national scale, compared to those with a subnational (n=19, 32%) or multinational (n=10, 17%) focus. All systems use some form of digital technology. Email communication or websites (n=40, 67%) and smartphones (n=29, 48%) are the most common technologies used, with some using both. Systems have capabilities to download geolocation data (n=31, 52%), photographs (n=29, 48%), and videos (n=6, 10%), and can incorporate lab data or sample collection (n=15, 25%). In sharing information back with users, most use visualization, such as maps (n=43, 72%); training and educational materials (n=37, 62%); newsletters, blogs, and emails (n=34, 57%); and disease prevention information (n=32, 53%). Out of the 46 systems responding to the survey regarding perceived impacts of their systems, 36 (78%) noted “improved community knowledge and understanding” and 31 (67%) noted “earlier detection.”

**Conclusions:**

The landscape demonstrated the breadth of applicability of participatory surveillance around the world to collect data from community members and trained volunteers in order to inform the detection of events, from invasive plant pests to weekly influenza symptoms. Acknowledging the importance of bidirectionality of information, these systems simultaneously share findings back with the users. Such directly engaged community detection systems capture events early and provide opportunities to stop outbreaks quickly.

## Introduction

The prevention and control of emerging pathogens relies on local-level surveillance for early detection of health events impacting people, animals, plants, and the environment. Many traditional animal and human health surveillance systems rely on data from astute clinicians, health center records, and laboratory testing to identify anomalies indicating potential outbreaks and emerging pathogens [1]. Plant surveillance systems also rely on laboratories, governmental systems, and border surveillance [2]. Unfortunately, access to health centers, laboratories, and veterinary services are not universally available to match the risk from emerging infections [3-5].

Exponential growth in accessibility to mobile technologies and web-based platforms across the globe has created unique opportunities for health surveillance systems to directly engage the public and key community stakeholders to rapidly collect data at the local level [1]. Direct engagement strategies have been employed across a spectrum of diseases to enhance disease surveillance [6]. The emergence of COVID-19 has led to a rapid increase in digital technology to directly engage the public in early detection and rapid response through apps for workplace health, contact tracing, and health information [7,8].

Participatory surveillance is defined as the bidirectional process of receiving and transmitting data for action through direct engagement of the target population. This approach can complement traditional surveillance systems by expanding engagement to larger segments of communities [1]. Participatory surveillance does not include data collected in a community for research or monitoring purposes when such data do not contribute to ongoing disease surveillance and do not provide information back to the community being monitored.

The evolution of participatory surveillance has been attributed to the use of participatory epidemiology in animal health, which recognized how local herdsmen and livestock owners have a deep knowledge of disease patterns and impacts among their livestock. Participatory epidemiology in animal health provides a co-learning opportunity between the animal health practitioner and the livestock owner that is built on trust and respect, in which practitioners interview livestock owners directly to understand disease burden among their herds. As such, this locally based surveillance data complements traditional animal health surveillance [3].

From animal health to human health, participatory surveillance integrates the locally based surveillance data to detect the emergence of an outbreak early, to expand surveillance capacity, and to inform control during outbreak response [3,9]. The earliest documented use of participatory surveillance in humans may have started with the “De Grote Griepmeting” in the Netherlands and Belgium in 2003 to monitor influenza-like illness [10]. Similar influenza-focused participatory surveillance systems now span several continents. Three International Workshops on Participatory Surveillance (IWOPS) have been held since 2013 to share best practices and explore innovative applications of this growing methodology [6].

COVID-19, Ebola virus disease, SARS, Middle East respiratory syndrome, and avian influenza outbreaks have highlighted the value of One Health in disease surveillance. One Health is a multisector approach that recognizes how the health of people, animals, plants, and the environment are inextricably connected [11,12]. For example, tracking changes in ecosystems helps identify potential areas for viral spillover [13]. With an estimated 60% of emerging infectious diseases being zoonotic in origin and 23% being vector-borne diseases, early detection among animal or vector hosts may limit or prevent outbreaks among people [12]. In addition to being vital components to health ecosystems, plants provide an estimated 70% of food sources to people and animals, thus ensuring that early detection of plant pathogens is critical for healthy environments, livestock, wildlife, and people [14].

Participatory surveillance systems vary in design and implementation. For human health, domesticated animal health, and crop health, participatory surveillance data are collected directly from the impacted individual, farmer, or rancher, or by a community or animal health worker on their behalf [15]. Wildlife and ecosystems provide a unique challenge as they do not have an “owner” to report, as would be expected for domesticated plants and animals. In these cases, the general public, trained volunteers, or land stewards, such as rangers, are typically called upon to assist in detecting morbidities and mortalities [16,17].

This landscape has been conducted to identify ongoing participatory surveillance systems across the One Health spectrum and to describe their geography, system logistics, and data and technology. Both single-sector and multisector systems are included in this landscape, as they both contribute to detection and response across the One Health spectrum.

## Methods

### Overview

Participatory surveillance systems for this landscape analysis were identified through existing partnerships, a systematic literature review, and surveys. Contacts of the systems identified through the literature review or ones known to the authors were sent a survey in order to collect the most up-to-date information where available. The survey link was also sent out through health organizations and to contacts of systems identified through stakeholder interviews in order to ensure that those systems not present in the peer-reviewed literature could be identified. Survey data and data abstracted from the literature were included in the analysis.

### Literature Review

In June 2021, a systematic literature review of English-language, peer-reviewed articles published after 1990 was performed using CAB Direct, PubMed, and the PubMed Veterinary Science search strategy. Preliminary search terms were developed based on input from the authors and were identified in an exploratory soft audit of phrases and words in an existing database of participatory surveillance articles using the text analysis tool Zotero 5.0 (Corporation for Digital Scholarship; Textbox 1).

**Search terms used in CAB Direct, PubMed, and the PubMed Veterinary Science search strategy for the literature review.**

**Search terms**

- “participatory epidemiology”
- “participatory surveillance”
- “participatory disease surveillance”
- “‘community-based surveillance’ or ‘community based surveillance’”
- “community event-based surveillance”
- “participatory” AND (“surveillance” OR “disease surveillance” OR “surveillance system” OR “disease detection”)
- (“crowdsourcing” OR “crowdsourced” OR “crowd-sourced” OR “crowd-sourcing”) AND (“surveillance” OR “disease surveillance” OR “surveillance system” OR “disease detection”)
- (“internet-based” OR “internet based”) AND (“surveillance” OR “disease surveillance” OR “surveillance system” OR “disease detection”)
- (“citizen science” OR “citizen-science”) AND (“surveillance” OR “disease surveillance” OR “surveillance system” OR “disease detection”)

Inclusion criteria for articles required them to describe participatory systems as (1) ongoing, (2) disease related, and (3) consistent with the definition of participatory surveillance [1]. A secondary reviewer conducted a review of all identified systems, potential systems, and a selection of the articles not meeting the inclusion criteria.

At the time of this landscape, COVID-19 surveillance systems were just beginning. While systems solely focusing on COVID-19 are not included in this paper, a future landscape could include those that have remained as ongoing surveillance systems and not as short-term response tools. Pilot systems were excluded unless it was clear that they were now ongoing systems.

To minimize inclusion of articles not relevant to ongoing, disease-related participatory surveillance, articles about COVID-19, maternal health, injuries, chronic conditions, drugs and smoking, and natural disasters were flagged. A character-by-character search of titles in R (version 4.1.1; R Foundation for Statistical Computing) flagged articles using the following terms: “covid|sars-cov-2|sarscov2,” “chronic|​diabet|crohn,” “maternal|fetal|natal|neonatal|pregnan|birth​ defect,” “injur|collision|bike|car,” “overdose|tobacco|smok|​vape,” and “natural disaster|storm|flood|drought.” Final exclusion or inclusion of the flagged articles was determined by manual review of the title and abstract.

Using RStudio (version 1.4.1717; RStudio, PBC) and R, resulting articles were concatenated into a single collection and dereplicated by title. A manual review of abstracts retrieved from PubMed through the Rentrez program in R showed additional noninfectious disease–related articles [18]. Thus, the following additional terms were used to remove nondisease-related articles: “cancer|oncolog|birth|obstretics|​violen|concussion.” As the survey was to be sent to any programs already identified by the authors, articles describing those systems were also removed from abstraction.

A total of 1584 articles were retrieved from PubMed and the PubMed Veterinary Science search strategy, and 851 articles were retrieved from CAB Direct. After deduplication, 276 articles were removed based on the exclusion criteria. During article abstraction, an additional 166 were categorized as not disease related, another 195 were not about participatory surveillance, and another 3 were related to COVID-19 and, hence, removed.

For each system meeting the inclusion criteria, reviewers collected the name, location, stated purpose, geographic scale, year the system began, number of users, technology used, types of data used, and how often users enter data. Abstraction also captured logistical information, such as identifying who enters the data, who analyzes and interprets the data, who maintains and operates the system, who responds to the data, and how the system is funded. Reviewers also captured whether data are specifically being used in forecasting or modeling, any challenges in implementation, and how challenges were addressed. Systems were categorized as human health, animal health, or environmental health. Human health systems were described by their target population. Animal health included the subcategories of livestock, equine, or poultry; wildlife other than birds; wildlife birds; fisheries; dogs or cats; or other animal species. Environmental health was subcategorized by the following focus areas: vector, waterborne, land use, food safety or food quality, crop, wild plant, or other. Multisector programs were identified as a combination of human health, animal health, or environmental health, as appropriate.

### Survey

Using Alchemer (version 5; Alchemer LLC), an online survey was conducted to capture data that were not identified through the peer-reviewed literature and to verify the data captured from the literature review. The survey collected the same data as those that were abstracted during the literature review, with additional questions related to syndromic and exposure data elements and whether a system had data freely available for public use. Staff piloted the survey to review clarity, usability, and time for completion. The Alchemer internal survey analysis tools noted high accessibility and low fatigue level for the survey tool.

In July 2021, the survey was sent using Mailchimp (Intuit) to the primary contact authors identified in the literature review, to contacts of systems that the authors had previously identified, and to those recommended by key contacts across the One Health spectrum. The survey was also sent out through the networks of the following: TEPHINET (Training Programs in Epidemiology and Public Health Interventions Network), EpiCore, the Wildlife Disease Association, Emory University’s Rollins School of Public Health and Environmental Health alumni, Johns Hopkins School of Public Health alumni, the National Plant Diagnostic Network, CORDS (Connecting Organizations for Regional Disease Surveillance), and the South Asia One Health Disease Surveillance Network.

### Ethical Considerations

The survey was not considered to be human subjects research, as data collected were limited to the organizational level for this landscape, which was designed to inform meeting planning for the fourth IWOPS; therefore, no ethics approval was applied for. This rationale is consistent with the Harvard University policies on human subjects research [19].

### Beta Review

Following survey data collection, reviewers reassessed which systems met the criteria to be considered as participatory surveillance systems. It was determined that bidirectional engagement requires feedback to be sent directly to the data-entering participants in order to inform them about the incidence of the event or any prevention or mitigation measures. Official government surveillance systems that were not bidirectional, including community health workers entering data into traditional surveillance systems, were excluded. Through this process, determinations were made to exclude those systems sharing data among veterinary professionals, as opposed to systems that collected data from paraprofessionals or volunteers from the general public. Those systems included the Small Animal Veterinary Surveillance Network, the Caribbean Animal Health Network, and Equinella, and they were excluded because such systems represented more of a traditional disease reporting system among health professionals.

### Data Analysis

To ensure the review contained the most up-to-date data available in this analysis, survey data were used where available; when they were not available, websites and literature review abstraction data were used. Descriptive data analyses were conducted in R and Microsoft Excel 2019 (version 2204).

## Results

### Overview

In total, 60 systems met the criteria for participatory surveillance (Multimedia Appendix 1) [15-17,20-57]. Systems were identified through the literature review (n=18, 30%) and from prior work by Ending Pandemics (n=21, 35%); the remainder were discovered through the survey outreach. The majority (n=43, 72%) of the programs discovered had a representative of the organization participate in the survey; information on the remaining programs (n=17, 28%) was gathered from available literature.

A total of 29 (48%) systems include data collected on human health, 26 (43%) include data collected on environmental health, and 24 (40%) include data collected on animal health. Of the 60 systems, 44 (73%) have a single-sector focus and 16 (27%) have a multisector focus (Table 1). Less than half of all systems self-identify as featuring a One Health focus (n=22, 37%).

Of the 44 single-sector programs, 22 (50%) only collect data on human health, 10 (23%) only collect data on animal health, and 12 (27%) only collect data on environmental health (Table 1) [16,17,20-23,25-31,35-39,45-52,56,57].

Among all 24 single-sector and multisector systems collecting data on animals, 16 (67%) only collect animal health data on wildlife, such as wild birds, mammals, reptiles, amphibians, and aquatics or fish [15-17,32-44,55-57]. In total, 8 (33%) systems collect data on domesticated animals, such as poultry, livestock, and companion animals. Only 1 (4%) system collects data on both wildlife and livestock animal populations [35].

Across the 26 systems that include data on environmental health, 16 (62%) collect data on vectors, 5 (19%) collect data on water quality, 3 (12%) collect data on food safety, 8 (31%) collect data on invasive species, 3 (12%) collect data on air quality, and 3 (12%) collect data on crops [15,32-34,40-54].

Most of the 29 systems collecting any data on human health look at multiple syndromes or diseases [26,27,29]. A total of 16 (55%) focus on influenza-like illness, with 12 (75%) of the influenza-focused systems expanded to incorporate COVID-19 surveillance. In total, 2 (7%) human health systems focus only on dengue.

Of the 16 multisector programs, the landscape found 2 (13%) systems focused on the combination of human and animal health, 9 (56%) focus on animal and environmental health, 2 (13%) focus on human and environmental health, and 3 (19%) focus on human, animal, and environmental health [15,32-34,40-44,53-55].

**Table 1:** Years in operation, geographic scale, and location of the participatory surveillance systems across the One Health spectrum.

Program^a^ focus	Programs (N=60), n (%)	Years in operation,^b^ mean (SD, range)	Geographic scale, n (%)				Continent, n (%)					
			Multinational or regional (n=10)	National (n=31)	Subnational (n=20)	Africa (n=4)		North America (n=16)	Asia (n=13)	Europe (n=18)	Australia (n=7)	South America (n=2)
Animal only	10 (17)	16.4 (11.8, 3-34)	2 (20)	5 (16)	3 (15)	0 (0)		5 (31)	1 (8)	2 (11)	2 (29)	0 (0)
Human only	22 (37)	7.5 (4.9, 1-17)	5 (50)	13 (42)	4 (20)	1 (25)		3 (19)	4 (31)	12 (67)	1 (14)	1 (50)
Environment only	12 (20)	8.4 (4.9, 3-20)	1 (10)	6 (19)	6 (30)	0 (0)		4 (25)	2 (15)	3 (17)	2 (29)	1 (50)
Human and animal	2 (3)	6.0 (0, 6-6)	0 (0)	2 (6)	0 (0)	1 (25)		0 (0)	1 (8)	0 (0)	0 (0)	0 (0)
Animal and environment	9 (15)	9.9 (4.7, 2-16)	2 (20)	4 (13)	3 (15)	1 (25)		3 (19)	2 (15)	1 (6)	2 (29)	0 (0)
Human and environment	2 (3)	7.5 (2.12, 6-9)	0 (0)	0 (0)	2 (10)	0 (0)		1 (6)	1 (8)	0 (0)	0 (0)	0 (0)
Human, animal, and environment	3 (5)	6.3 (1.5, 5-8)	0 (0)	1 (3)	2 (10)	1 (25)		0 (0)	2 (15)	0 (0)	0 (0)	0 (0)

^a^Data were from 60 participatory surveillance systems identified through the systematic literature review and surveys.

^b^The mean years in operation for all programs combined was 9.3 (SD 6.8, range 1-34).

### Geography of the Systems

Out of the 60 identified systems, approximately half (n=31, 52%) are designed to be used at a national scale, whereas one-third (n=20, 33%) have a subnational focus and one-sixth (n=10, 17%) have a multinational focus [17,26,27,29,35,36,45,46,49-53,56,57]. Among the 44 single-sector systems, 24 (55%) have a national focus. Out of the 16 multisector systems, 7 (44%) have a subnational geographic focus.

Only 4 systems out of 60 (7%) were identified from Africa. The remaining systems came from the Americas (n=18, 30%), Asia (n=13, 22%), Australia (n=7, 12%), and Europe (n=18, 30%; Table 1) [17,26,27,29,35,36,45,46,49-53,56,57]. Many of the multinational systems span the United States, Canada, Mexico, the United Kingdom, or Europe. Another multinational system is AVADAR (Audio-Visual Acute Flaccid Paralysis Detection and Reporting), which covers Nigeria, Sierra Leone, Liberia, the Democratic Republic of the Congo, Chad, Niger, South Sudan, and Cameroon [20]. WildHealthNet and SMART (Spatial Monitoring and Reporting Tool) for Health are used to provide actionable data on a national and subnational basis across Cambodia, Laos, and Vietnam.

### System Logistics

When asked to describe who enters the data, who responds to the data, how often those data are entered, and what feedback is provided back to the end user, systems reported a range of user types. For the 22 systems that only focus on human health, almost all (n=18, 82%) list the general public as the user; the remaining users are trained volunteers and health care workers [26,27,29]. Of the 10 systems that only focus on animal health, 5 (50%) rely only on the general public, 2 (20%) use trained volunteers and the general public, 1 (10%) uses trained volunteers, 1 (10%) uses wildlife rehabilitators, and 1 (10%) uses farmers and rangers [17,35,36,56,57].

When asked to categorize the number of users by range (from <500 to >50,000), 16 out of 60 (27%) systems reported having under 500 users, 14 (23%) reported having 500 to 5000 users, and 12 (20%) reported having 5000 to 15,000 users [26,27,29,35,36,45,46,50-52]. A total of 3 systems out of 60 (5%) reported having more than 50,000 users: FluTracking from Australia, California’s West Nile Virus surveillance system, and Cambodia 115 Hotline [17].

Across the 60 identified systems, the user determines when to report in over half of the systems (n=34, 57%), including in 7 out of the 10 (70%) systems focused on only animal health, 10 of the 12 (83%) systems focused on environmental health, 14 of the 16 (88%) multisector systems, and only 3 of the 22 (14%) human health systems [17,26,27,29,35,36,45,46,49-53,56,57]. Out of the 23 programs using weekly reporting, 19 (83%) were systems that collect data only on human health [26,27,29,53]. The weekly reporting systems included the influenza surveillance systems, DoctorMe, Participatory One Health Digital Disease Detection, Kidenga, Egypt’s Community-Based Animal Health Outreach (CAHO) surveillance system, and AVADAR [26,27,29,53]. DoctorMe, iMammalia, and Brazil’s Guardians of Health reported that data are collected daily. FeederWatch reports are limited to November to April, when the greatest amount of bird feeder activity occurs [36]. Similarly, Mozzie Monitors, a mosquito surveillance system, focuses its reporting during peak mosquito season for the presence of vectors [46]. Outbreaks Near Me noted that they prompt users every 3 days by SMS.

A total of 42 systems stated that once data are reported, response is led by government or academic institutions [17,26,27,29,35,36,45,46,49-53,56,57]. Nonprofits are primary responders for 11 of these systems (26%), 6 of which (55%) are systems that collect both animal and environmental data. Private sector partners are responders for 5 (13%) systems [35]. Government response agencies usually include health, environment, or agriculture agencies. iMammalia shares data with the Food and Agriculture Organization of the United Nations (FAO) as appropriate, and Outbreaks Near Me shares trends with government agencies. Guardians of Health specified that schools and universities are involved in the response.

### Data and Technology

All the systems in this analysis use some form of digital technology, with the exception of Egypt’s CAHO, though 6 out of 60 (10%) still incorporate paper-based data collection. Email communication or websites (n=40, 67%) and smartphones (n=29, 48%) are the most common technologies used [17,26,27,29,36,45,46,49,51-53,56,57], with several systems using both. Smartphones are often used for collecting environmental health data only [45,46,49,51,53]. All systems that collect data only on human health use email or web-based systems [26,27,29]. The Ukraine Infectious Diseases of Animals system was the only program stating that they incorporate remote sensing. Cambodia 115 Hotline is the only system that reported using voice recording, also known as interactive voice response.

Many of the 60 systems have the capability to upload geolocation (n=31, 52%) and photographs (n=29, 48%); a few are able to upload videos (n=6, 10%) [17,35,36,45,46,49,51,52,56,57]. A total of 12 out of the 15 (80%) systems incorporating lab testing or diagnostics focus on animal health or environmental health; many collect carcass or vector samples [35,46,50,51]. The Cervid Disease Network often anonymizes location to protect the end user and their farms [35]. A total of 20 systems (33%) use data in forecasting or modeling [17].

Out of 43 systems that answered, a total of 11 (26%) survey respondents answered that their data are publicly available. In total, 21 (49%) systems reported that their data are not openly available. In addition to those 21 systems, 3 (7%) stated that data are held by government agencies and not available to the public; 8 (19%) systems specified that data are sometimes available in summary format or at the request of the researchers, but with redaction of any protected information.

Bidirectionality (ie, providing information back to the users) is essential for meeting the criteria for participatory surveillance. Systems (N=60) share information back to users in a variety of ways, including visualization, such as maps (n=43, 72%); training and educational materials (n=37, 62%); newsletters, blogs, and emails (n=34, 57%); and disease prevention information (n=32, 53%; Table 2) [17,26,27,29,36,45,46,49,51-53]. Vaccine information is shared back by 8 (13%) systems, and 5 (63%) of these have a human health focus. Treatment and medical advice (n=16, 27%) is also provided [45,46,51,53]. Users are provided with disease data by 16 (27%) systems [17]. FishWatch and PestWatch systems provide information back through the media and trained volunteers [56,57]. The California Wildlife Morbidity and Mortality Event Alert System noted that staffing constraints limit when they are able to respond directly back to a report. The Arizona Game and Fish Department specified that they report findings and results back to the users.

Survey respondents were asked to note all of the impacts their systems have had to date (Table 3). Out of the 46 systems that reported through the survey, 36 (78%) noted that “improved community knowledge and understanding” was an impact of their system and 31 (67%) stated that “earlier detection” was an impact of their system. In the text field, 1 (2%) system wrote “improved active surveillance” and another (n=1, 2%) wrote “slowly improving stakeholder and partner understanding.” Survey respondents were not asked to justify or provide examples of these impacts.

**Table 2:** Information about participatory systems that reported providing information back to users.

System^a^ focus	Information provided by systems,^b^ n (%)						
	Visualization of the situation^c^ (n=45 )	Disease data from other sources (n=16)	Vaccine information (n=8)	Disease prevention information (n=34)	Treatment or medical advice (n=16)	Newsletters, blogs, or email updates (n=35)	Training or educational materials (n=40)
Animal only	5 (11)	4 (25)	0 (0)	4 (12)	1 (6)	6 (17)	8 (20)
Human only	18 (40)	5 (31)	5 (63)	9 (26)	3 (19)	17 (49)	8 (20)
Environment only	10 (22)	1 (6)	0 (0)	9 (26)	3 (19)	6 (17)	12 (30)
Human and animal	1 (2)	0 (0)	0 (0)	1 (3)	0 (0)	0 (0)	0 (0)
Animal and environment	6 (13)	4 (25)	1 (13)	6 (18)	5 (31)	5 (14)	8 (20)
Human and environment	2 (4)	0 (0)	0 (0)	2 (6)	2 (13)	0 (0)	2 (5)
Human, animal, and environment	3 (7)	2 (13)	2 (25)	3 (9)	2 (13)	1 (3)	2 (5)

^a^Data were from 60 participatory surveillance systems identified through the systematic literature review and surveys.

^b^Links to public health resources were not provided by any of the systems.

^c^Visualization included maps of cases.

**Table 3:** Self-reported impacts of participatory surveillance systems.

System^a^ focus	Impacts of systems, n (%)					
	Earlier detection (n=31)	Improved community knowledge and understanding (n=36)	Quicker response (n=26)	Better cross-sector coordination (n=25)	Policy or funding impacts (n=19)	Have not measured impacts (n=6)
Animal only	5 (16)	5 (14)	5 (19)	4 (16)	3 (16)	0 (0)
Human only	11 (35)	16 (44)	7 (27)	11 (44)	5 (26)	2 (33)
Environment only	3 (10)	4 (11)	2 (8)	1 (4)	1 (5)	1 (17)
Human and animal	2 (6)	1 (3)	2 (8)	1 (4)	1 (5)	0 (0)
Animal and environment	7 (23)	7 (19)	7 (27)	5 (20)	6 (32)	2 (33)
Human and environment	1 (3)	1 (3)	1 (4)	1 (4)	1 (5)	0 (0)
Human, animal, and environment	2 (6)	2 (6)	2 (8)	2 (8)	2 (11)	1 (17)

^a^Data were provided by 46 of the programs through the survey.

## Discussion

### Principal Findings

Across the One Health spectrum, participatory surveillance is being used around the globe to improve animal, human, and environmental health. The majority of the systems in this paper were identified through the survey outreach, suggesting that many systems have not yet been described in the peer-reviewed, English-language literature. As such, numerous additional systems may exist that have not been captured within this assessment. As a complement to this manuscript, an updateable digital map will be made available to the public to provide a repository of the systems identified in this landscape and to provide a platform to add in new systems as they are identified.

Many systems reported impacts of improved early detection and quicker response. Trained volunteers and members of the public augment current disease surveillance activities of health department staff to engage larger populations and expand geographic coverage. Thus, such systems may identify events when traditional systems would not have the personnel or other resources to detect early. For example, faced with the challenge of identifying invasive species of plant pathogens and insect pests across the 244 million acres of cultivated crops and 640 million acres of federally managed public lands in the United States, the University of Georgia’s participatory surveillance system captures data from over 30,000 professionals and trained volunteers, through the EDDMapS (Early Detection and Distribution Mapping System) [41], and from the public, through Wild Spotter. Data collected are able to inform official systems, such as the National Plant Diagnostic Network, so they may follow up with an appropriate response [58].

Nonprofits were noted to play a larger role in response regarding animal health and environmental health systems compared to human health; further assessment would be required to understand if this is due to their role in funding of the initial systems or due to limited government response capabilities in these fields.

Confirming bidirectionality was a challenge for wildlife and wildland surveillance, where there is not necessarily a direct ongoing link between the user and the impacted plant or animal. Numerous systems were identified that collect data from persons witnessing a change in a landscape, a dead animal, or a vector, but they did not specify whether information was reported back to the individuals; hence, they were not included in this analysis. In contrast, other wildland networks have incorporated repeated reporting of site locations to gather both negative report data and recurring records. One such case is the inclusion of a sentinel tree program by the United Kingdom group Observatree, where users selected a single tree or group of trees to report on their health and any changes in conditions several times a year [52]. While systems that rely solely on single event–based reporting with no additional follow-up were excluded from this landscape, they demonstrate distinct differences in how data are collected in wild versus domesticated situations, and the need to consider how to encapsulate such systems in the broader participatory surveillance landscape.

Often, systems with more users were those that collected data from the general public. Human health systems, many of which were based on a similar framework for influenza surveillance, were most likely to require regular reporting intervals. It will be interesting to see if changes in seasonal practices of reporting for birdfeeder and vector systems will be needed as climate change impacts migratory and weather patterns. This effort did not collect data on recruitment and sustainability; these would be worth exploring in future studies as well.

Impact data suggest that systems perceive they are meeting their stated goals for early detection, response, and outreach. Many animal and human health systems reported early detection and rapid response as outcomes from their systems. Further data collection through interviews and review of monitoring and evaluation systems would be required to assess and quantify impacts and to understand why fewer environmental systems had seen these impacts.

Multisector data collection and integration provide both challenges and opportunities to enabling a One Health approach to detection and response. Siloed government systems, data sharing challenges, different professional terminologies, and priorities create obstacles to developing multisector systems that capture human, animal, and environmental health data.

However, 14 multisector systems identified across 4 of the 5 OIE (World Organisation for Animal Health; formerly, Office International des Epizooties) regions have been active anywhere from 2 to 16 years. Conceivably, it may be easier to integrate data collection at the local level through participatory systems simply because the animals, people, and plants are geographically colocated. A next step in reviewing this dynamic should include assessing processes for data integration and interoperability among multisector participatory surveillance and discerning how those data can be used to inform potentially separate formal systems.

Innovations in technology are enhancing capabilities in capturing data from the public, from geolocation, to video, to sample collection. Pairing of laboratory data and point-of-care diagnostics with participatory surveillance systems may add to the specificity of this approach.

Emerging wearable technologies are creating new diagnostic capabilities for plants, animals, and people; these may continue to enhance the specificity of data collected from participatory surveillance [59-61]. In terms of geolocation data collection, the anonymization approach of the Cervid Disease Network may be worth considering for other livestock or crop surveillance systems for which concerns about farm identification may deter participation [35].

### Limitations

Selection bias may have skewed results based on outreach conducted through networks that may not encompass all systems. The survey identified numerous programs that were not identified in the literature review, indicating that not all systems are discoverable in the English-language peer-reviewed literature. Systems developed for limited duration focusing on a single outbreak response were not included. It is possible that such systems, like COVID-19 monitoring or new pilot efforts, will become long-term systems and may need to be included in the future. The literature review and survey were conducted exclusively in English, which also likely undercounted the number of systems that are currently active. In fact, one survey was not complete enough to include, and the limited answers that were provided were not in English. The authors will continue their discovery of systems; in addition, any new systems uncovered by the authors can be included in the interactive map that is under development and that will be made publicly available online at the Ending Pandemics website [62].

Incomplete data from respondents limited this study’s abilities to interpret findings across all systems. In addition, the fact that literature data were used when survey data were not available may have prevented inclusion of the most up-to-date information for those systems. Systems that began after July 2021 were not included in this analysis.

In 2017, Ending Pandemics published a landscape of participatory surveillance systems based on partnerships it had with other system developers through its convenings at the IWOPS. A loose collaboration of participatory surveillance system creators and stewards, IWOPS partners met for the first time in 2012 in San Francisco, United States; again in 2013 in Amsterdam, the Netherlands; and most recently in 2016 in Newcastle, Australia. IWOPS serves as an informal network to share best practices, consult on analytic methods, and catalyze innovations to advance the direct engagement of populations in voluntary reporting. The 2017 review was limited to IWOPS partner systems and revealed 23 distinct participatory surveillance tools or programs in 18 countries that encompassed human and animal health [6]. This study summarized results from the systematic review of the literature combined with a detailed survey of all identified systems in human, animal, and environmental health. While prior work focused on a convenience sample, this landscape incorporated literature review and survey methodology. This landscape also incorporated systems that only focus on plant health and environmental health.

### Conclusions

This landscape demonstrated the breadth of applicability of participatory surveillance, from tick identification in photographs, to One Health apps used by community members, to trained volunteers reporting invasive plant pests, to people tracking their own weekly influenza symptoms around the world. With globalization, trade, and travel, rapid disease spread across country borders creates a need for on-the-ground detection systems that can capture cases early and provide opportunities to stop outbreaks quickly. Developing mechanisms for information sharing among participatory surveillance systems may improve opportunities for systems to alert others as to what may be on the horizon. These actions may require revisiting ways to allow for public data access and sharing in formats that protect sensitive data.

In this review, some systems demonstrated the importance of being in place in advance of a pandemic, as they were able to be easily adapted for information collection and communication with the public specific to COVID-19. As the World Health Organization—in conjunction with the FAO, the OIE, and the United Nations Environment Programme—develops the Epidemic Intelligence from Open Sources platform, finding timely ways to integrate participatory surveillance data will be critical. For the hundreds of thousands of participatory surveillance users, seeing a global, as well as a local, impact of their efforts may help inspire them to continue in these voluntary roles.

## Appendix

Multimedia Appendix 1 Supplementary table of data from participatory surveillance systems.

## Abbreviations

AVADAR: Audio-Visual Acute Flaccid Paralysis Detection and Reporting
CAHO: Community-Based Animal Health Outreach
CORDS: Connecting Organizations for Regional Disease Surveillance
EDDMapS: Early Detection and Distribution Mapping System
FAO: Food and Agriculture Organization of the United Nations
IWOPS: International Workshop on Participatory Surveillance
OIE: World Organisation for Animal Health; formerly, Office International des Epizooties
SMART: Spatial Monitoring and Reporting Tool
TEPHINET: Training Programs in Epidemiology and Public Health Interventions Network
